# A New Few-Shot Learning Method of Bacterial Colony Counting Based on the Edge Computing Device

**DOI:** 10.3390/biology11020156

**Published:** 2022-01-19

**Authors:** Beini Zhang, Zhentao Zhou, Wenbin Cao, Xirui Qi, Chen Xu, Weijia Wen

**Affiliations:** 1 Advanced Materials Thrust, Department of Physics, The Hong Kong University of Science and Technology, Hong Kong; bzhangay@connect.ust.hk; 2 Clearwaterbay Biomaterials Ltd., Shenzhen 518100, China; zhouzt@xbiomaterials.com (Z.Z.); caowb@xbiomaterials.com (W.C.); 3 Department of Physics, The Hong Kong University of Science and Technology, Hong Kong; xqiah@connect.ust.hk (X.Q.); cxuba@connect.ust.hk (C.X.)

**Keywords:** few-shot learning, bacterial colony counting, edge computing

## Abstract

**Simple Summary:**

Here, we proposed a few-shot learning bacterial colony detection method based on edge computing devices, which enables the training of deep learning models with only five raw data through an efficient data augmentation method.

**Abstract:**

Bacterial colony counting is a time consuming but important task for many fields, such as food quality testing and pathogen detection, which own the high demand for accurate on-site testing. However, bacterial colonies are often overlapped, adherent with each other, and difficult to precisely process by traditional algorithms. The development of deep learning has brought new possibilities for bacterial colony counting, but deep learning networks usually require a large amount of training data and highly configured test equipment. The culture and annotation time of bacteria are costly, and professional deep learning workstations are too expensive and large to meet portable requirements. To solve these problems, we propose a lightweight improved YOLOv3 network based on the few-shot learning strategy, which is able to accomplish high detection accuracy with only five raw images and be deployed on a low-cost edge device. Compared with the traditional methods, our method improved the average accuracy from 64.3% to 97.4% and decreased the False Negative Rate from 32.1% to 1.5%. Our method could greatly improve the detection accuracy, realize the portability for on-site testing, and significantly save the cost of data collection and annotation over 80%, which brings more potential for bacterial colony counting.

## 1. Introduction

Bacterial Colony Counting (BCC) is a time consuming but important task for many fields such as microbiological research, water quality monitoring, food sample testing, and clinical diagnosis [1,2,3]. The predominant need for these applications is accurate quantification, and with the growing problems of microbial contamination, the need for on-site testing is also increasing daily [4,5,6]. Fast and accurate on-site testing can reduce the cost of transporting samples and decrease the risk of leakage and further contamination [7], which is important for the management of contaminants [8]. To achieve accurate counting, image analysis methods play the most important role in BCC, and there are three main types for the quantification now: manual counting, traditional image segmentation algorithms, and deep neural networks [9]. The bacterial colonies are difficult to be recognized when directly cultured on solid agar plates because they have the features of high density, low contrast, adherence, and overlap [10]. So, manual counting is still the gold standard for BCC because of the high precision, but manual counting is quite time consuming and cannot be adapted to high throughput industrial testing [2]. Traditional algorithms such as threshold segmentation, watershed, and wavelet transform provide possibilities for automation recognition, but they face difficulty in processing images with low contrast and a complicated overlap situation [11,12]. On the contrary, deep learning networks based on convolution neural networks (CNN) are good at dealing with complicated problems [13,14]. However, most deep neural networks are designed to be deployed on professional deep learning workstations, which have a high requirement for device configuration [9,15,16]. However, professional workstations are expensive and bulky, making them difficult to meet the requirement of portability, and for many application scenarios such as remote reservoirs and farms, samples can only be taken back to the laboratory for colony testing, which has a great delay and increases the cost of sample transportation [17,18,19]. In addition, the training of deep neural networks usually requires a large number of data, but there is no large public dataset of bacterial colony images at present, so the cost of data collection and annotation would be high if the traditional deep learning strategy was adopted [20,21,22,23].

To solve these problems, we propose a new few-shot learning method that consists of the improved You Only Look Once (improved YOLOv3) for image detection and Random Cover Targets Algorithm (RCTA) for data augmentation. The improved YOLOv3 adopted multi-scale features for object detection through the Feature Pyramid Network (FPN), which effectively improves the detection accuracy for small targets. Therefore, our method does not require special equipment such as colored Petri dishes or high-resolution cameras to enhance the contrast, which greatly reduces the detection costs. On the other hand, the training of most neural networks usually requires a large number of training images, but colony images are expensive to collect and time consuming to label. The RCTA proposed in this paper utilized the prior knowledge of bacterial colony images to solve this dilemma, which could effectively increase the data to more than 300 times after combining with cutting and rotation operations. Thus, our network was able to be successfully trained by only five raw data and achieved the high accuracy of 97.4% on an edge computing device that cost less than USD 100. Our method greatly reduced the cost of data collection and annotation, increased the detection accuracy, and met the portability requirement of on-site testing. In addition, with TesnorRT acceleration, our model could achieve all detection on edge computing devices locally, eliminating the need to interact with the cloud for data transmission, which reduced the dependence of network and costs significantly.

## 2. Materials and Methods

### 2.1. Data Preparation and Materials

The strain used in this experiment was *Escherichia coli* (ATCC8739) [24,25,26,27], which was purchased from Guangdong Huankai Microbiology Technology Co Ltd. The Tryptic Soy Broth (TSB) was used as the liquid medium [28], the Plate Count Agar (PCA) was used as the solid medium [29], and the 0.9% sterile saline was used as the dilution solution [29,30]. The steps of colony culture are: first, inoculate the strain into 100ml TSB medium and incubate it at 37 °C and 200 rpm for 20 h to obtain the bacterial solution [24]; second, according to the Chinese National Standard [29], dilute the bacterial solution with saline to 30–300 CFU/mL, take 1 mL diluted bacterial solution and mix it with 15 mL PCA, then place it at 37 °C for 48 h.

### 2.2. Equipment

**Figure 1:**
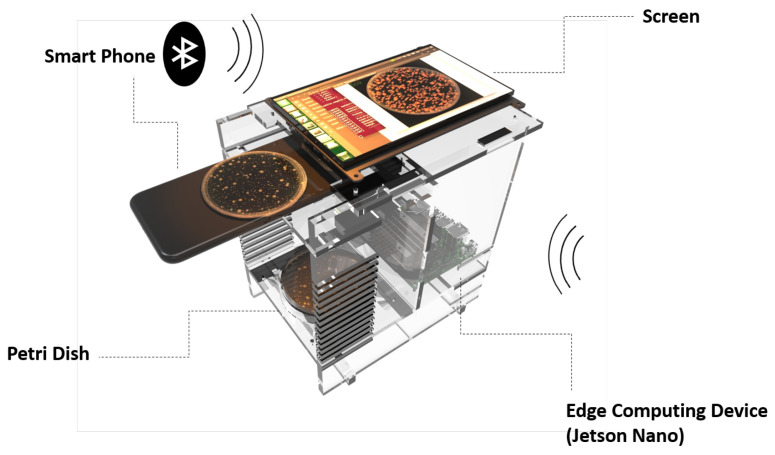
The prototype of bacteria counting based on the edge computing device. The Petri dish contains the bacterial colonies, the smartphone is the photo device, and the data are transmitted to the jetson nano via Bluetooth. The jetson nano takes responsibility for the image detection, and the screen is responsible for visualizing the detection results.

The training of the models was performed on the deep learning workstation with an Intel Core I7-9800X processor and 2 GeForce RTX 2080 Ti graphics cards. The testing of the models was performed on a microcomputer jetson nano, which had a 128-core NVIDIA Maxwell™ GPU and 4GB of 64-bit LPDDR4 memory. The prototype of our portable BCC device based on deep learning method is shown in Figure 1. Most detection devices of BCC require professional cameras with high resolution to enhance the contrast and thus improve the algorithm detection accuracy. Considering the mobility and portability of detection, we choose the smartphone rather than professional cameras to afford photographs. The experimental images in this paper are all taken by the Sony IMX586. The camera of IMX586 has 48 megapixels with a resolution of 8000 ∗ 6000 (width ∗ height) and 0.8 μm per pixel, and its field of view is $88^{\circ }$.

### 2.3. Dataset

The dataset used in this paper can be divided into three types: train, validation, and test. The train and validation datasets were used to train the model, which contained 864 images and 96 images, respectively. All images of the train and validation dataset were augmented by 5 original pictures, that of 2560 ∗ 2590 (width ∗ height) pixels. During the augmentation procedure, we first used RCTA to randomly cover targets in each image, which expanded the number of images to 60. Secondly, we divided the image into four equal parts, which could magnify the relative proportion of targets and effectively augment the data four times. For the final augmentation, we adopted three rotation operations: 90 degrees, 180 degrees, and 270 degrees. Finally, we obtained 960 images of 1280 ∗ 1290 (width ∗ height) pixels. These pictures were randomly allocated as the ratio of 90% and 10% to form the training dataset and validation dataset. As for the test dataset, the main function of it was to measure the performance of trained models. So, we carried out 60 colony culture experiments to obtain 60 completely new images that were independent of the train and validation dataset. Then, we randomly selected 10 images to compare the performance of four models: simple threshold, comprehensive threshold, tiny YOLOv3, and improved YOLOv3.

### 2.4. Method Overview

**Figure 2:**
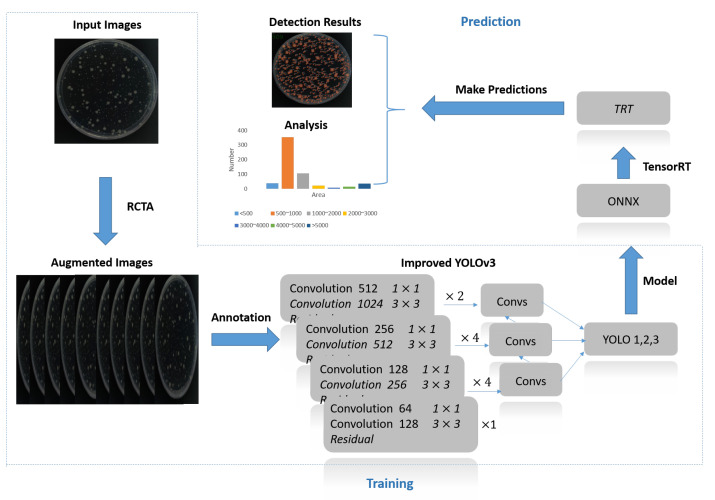
Method overview. The input image is first augmented by RCTA, then bacterial colonies need to be manually annotated. The annotation files and the augmented images will be used as the input of improved YOLOv3 for training. The trained model needs to be converted to the intermediate ONNX format first and then converted to the TRT format that can be deployed on jetson nano with high processing speed. The colony detection and analysis will be performed by the converted model.

Our method can be mainly divided into two stages as Figure 2 shows: training and prediction. Since there is a high cost attached to data collection and annotation, it is quite expensive to collect a large amount of dataset for BCC. So, we first need to effectively augment the original data by Random Cover Target Algorithm, which is proposed in Section 2.5. By rewriting the pixel values of target points, RCTA could change the structure of images and make the target points decrease regularly as iterations increase. After adopting RCTA, only the source images need to be fully manually annotated. For augmented images, RCTA would copy the annotation file of the source file, and we only need to remove the redundant labels rather than repeatedly mark all targets, which can greatly reduce the annotation cost by over 80%. With this method, the improved YOLOv3 could successfully achieve the few-shot learning with only 5 original training images and accuracy over 95%.

After successfully training the model with reliable accuracy through the darknet framework, we need to deploy the model on the embedded device for ensuring the portability of BCC. However, since the hash rate of embedded devices is much lower than workstations, we need to optimize the model by TensorRT to guarantee the processing speed. Since TensorRT does not support darknet models directly, we need to convert the trained model and weights into .onnx format at first. Then, TensorRT will further convert the model to trt format by building an inference engine that optimizes the CNN networks of the improved YOLOv3 through precision calibration, interlayer merging, and dynamic memory management. After optimization, due to the reduction in the number of data transfers between layers and the narrowing of the data precision range, the processing speed of the model will be greatly improved. In addition, by precisely positioning the detection boxes, we can calculate the length, width, and size of each bacterial colony. The detection boxes of the improved YOLOv3 contain two sets of information: ($x_{1}$,$y_{1}$) and ($x_{2}$,$y_{2}$), which represent the coordinates of the top left and bottom right points of the detection box, respectively. So, the width of the bacterial colony is $x_{2}-x_{1}$, height is $y_{2}-y_{1}$, and area is width*height. With these data, we are able to count the size interval and number distribution of the corresponding bacterial colonies as shown in the analysis of Figure 2.

### 2.5. A New Data Augmentation Method

In recent years, deep neural networks have made great improvements in the field of target recognition, solving many complex problems that are difficult to be processed by ordinary algorithms. However, deep neural networks require a large number of labeled images for training, and the annotation cost is pretty high for targets with complex features or rare datasets [31]. Few-shot learning strategy can solve the problems of insufficient data and high cost of labeling through effective data augmentation methods, and this strategy is become an increasingly important branch of deep learning.

For bacterial colony images, the targets are relatively small and the number of colonies for a single image usually ranges from hundreds to thousands. Additionally, even under the condition of heating catalysis, the culture time of bacterial colonies lasts hours. So, it is difficult to obtain a large number of data, and the manual labeling method for traditional deep learning networks is quite time consuming. Therefore, we proposed a data augmentation method, Random Cover Targets Algorithm, to achieve effective few-shot learning with lower cost in this paper.

Functionally, RCTA realized the data augmentation by accurately changing the pixel values of the target areas to the values of background, which could change the structure of images and make them into new images. To achieve the above functions, RCTA would first use threshold segmentation to initially segment the background and effective targets. Then, we used the cv2.findContours() function to identify the contour of effective targets, which aimed to obtain the area of each recognition area and set the approximate effective range for secondary selection. The effective range was usually decided by past experience and manual fine-tune, and it was (60, 3500) in this experiment. For secondary selection, only the areas that were among an effective range would be kept. Third, RCTA would choose the central area that excluded the boundary area as the final coverage area. For the effective points in the coverage area, RCTA would store their (x,y) coordinates and radius into the array, and it randomly selected one at a time as the parameter of cv2.circle() function. Finally, in order to blend the target area with the surrounding background as much as possible, we used the average pixel value of $\left(x_{r}:x_{r+20},y_{r}:y_{r+20}\right)$ to calculate the $R_{mean}$, $G_{mean}$, and $B_{mean}$. According to the gray value analysis, the pixel value of the background area was usually lower than 20, so we only chose the pixel values that were lower than 20 for calculation. The calculation method is as follows:

(1) $$R_{mean}=\sum_{x=x_{r},y=y_{r}}^{x=x_{r+20},y=y_{r+20}}R_{\left(x,y\right)}/N$$

(2) $$G_{mean}=\sum_{x=x_{r},y=y_{r}}^{x=x_{r+20},y=y_{r+20}}G_{\left(x,y\right)}/N$$

(3) $$B_{mean}=\sum_{x=x_{r},y=y_{r}}^{x=x_{r+20},y=y_{r+20}}B_{\left(x,y\right)}/N$$

(4) $$\left[\begin{array}{c} x_{R}\\ y_{R}\\ 1 \end{array}\right]=\left[\begin{array}{c} x_{o}\\ y_{o}\\ 1 \end{array}\right]\left[\begin{array}{ccc} \cos\left(\theta \right) & -\sin\left(\theta \right) & 0\\ \sin\left(\theta \right) & \cos\left(\theta \right) & 0\\ -0.5w\cos\left(\theta \right)-0.5h\sin\left(\theta \right)+0.5W & -0.5w\sin\left(\theta \right)+0.5h\cos\left(\theta \right)+0.5H & 1 \end{array}\right]$$

In Formulas (1)–(3): $\left(x_{r},y_{r}\right)$ represent the coordinates of the bacterial colonies centers; $R_{mean}$, $G_{mean}$, and $B_{mean}$ represent the mean value of the red, green, and blue channels; $R_{\left(x,y\right)}$, $G_{\left(x,y\right)}$, and $B_{\left(x,y\right)}$ represent the pixel values that are lower than 20 in the calculation area; N represents the number of pixels. In the actual experiments, the brightness value of the background is not related to the surrounding environment since the photography of BCC is usually carried out in a shading environment to avoid reflections of light spots. So, the background brightness of BCC is relatively uniform, and the average value of adjacent pixels can achieve a good coverage effect. Formula (4) is the calculation principle for rotation operations, where $x_{o}$ and $y_{o}$ represent the coordinates of the original image; $x_{R}$ and $y_{R}$ represent the coordinates after rotation; h and w represent the height and weight of original image, respectively; H and W represent the height and weight of rotation image; θ represents the rotation angle. For our experiments, we adopted three angles for rotation, which are $\theta =90^{\circ },180^{\circ },270^{\circ }$.

**Figure 3:**
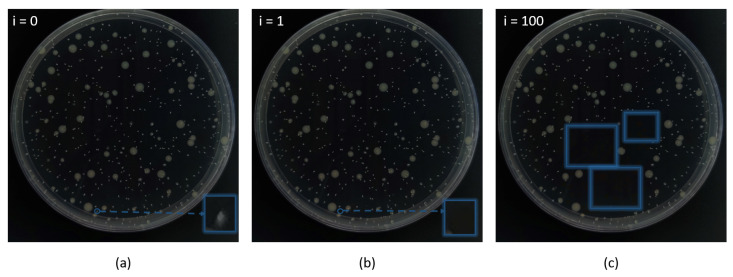
Data augment result. (**a**) Original image. (**b**) Iteration = 1. (**c**) Iteration = 100. RCTA will achieve different numbers of data augmentation by adjusting iteration, and i represents the number of iteration. With i increases, the number of colonies whose pixel values are changed by RCTA will also increase, thus changing the image structure.

**Figure 4:**
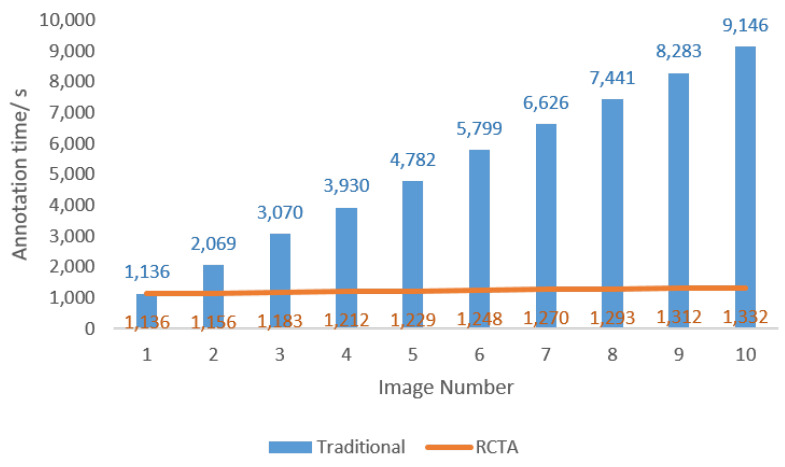
Annotation time performance comparison between RCTA and traditional annotation method. The blue bar represents the time used for the traditional annotation method, and the orange line represents the time used for the RCTA annotation method. The traditional method needs to manually annotate all targets, but RCTA only needs to remove the redundant annotation boxes for augmented images, so the annotation time of RCTA is reduced by over 80% compared to the traditional method as the number of images increases.

Due to the low brightness and weak contrast of the colony, the threshold segmentation and cv2.findContours() function can only find limited targets among the image, which cannot be used as the precise quantitative method. However, they can effectively provide a benchmark for the background transfer procedure in RCTA. The example of the RCTA augmentation result is shown in Figure 3, through effective adjustment of parameters and iterations, RCTA can amplify an original picture to more than hundreds, which can hugely decrease the demand for the original training dataset. In addition, the annotation time comparison between the traditional annotation method and RCTA is shown in Figure 4. Traditional annotation methods require fully manual annotation for each image, which is time consuming because the colony images usually contain a large number of targets. For this example, the annotation time for a single image using the traditional method is typically over 18 min. With RCTA, we only need to manually annotate the source image, and the subsequent augmented images will copy the annotation file of the source image, so that only redundant boxes after masking need to be removed, which could greatly reduce the average annotation time by over 80%.

### 2.6. Training Strategy

Since most CNN models are not sensitive to small targets, for the previous works that do not adopt any pre-process to increase the contrast between culture dish and bacteria, their recognition rate for small targets is relatively poor [9]. Although YOLOv3 improves the detection performance for small targets by dividing the grids at three different scales, which aims to predict the contour of the targets falling into the grid through different densities and receptive fields, due to the extremely small size of the dotted bacterial colonies, they are still difficult to recognize directly.

To solve this problem, we experimented a scaling mapping strategy between cutting images and original images. By reducing the length and width of the image, we could increase the relative size of the object. In our experiment, we divided the original image into four equal parts so that the width and height of each part were reduced from 2560 ∗ 2590 pixels to 1280 ∗ 1295 pixels. Cutting images off did not change the absolute length of the bacterial colonies, but due to the decrease in image size, the relative size ratio of the bacterial colonies would be increased by two times. The corresponding relationship between the bacterial colonies’ diameters before and after cutting is shown in Formula (4):

(5) $$\frac{L_{cut}}{W_{cut}}=\sqrt{D}*\frac{L_{original}}{W_{original}}\;\left(D=4,9,16\ldots \right)$$

where $L_{cut}$ and $L_{original}$ represent the length of the bacterial colony, $W_{cut}$ and $W_{original}$ represent the width of the image, and D represents the number of division parts for the image.

### 2.7. Structure and Acceleration

**Figure 5:**
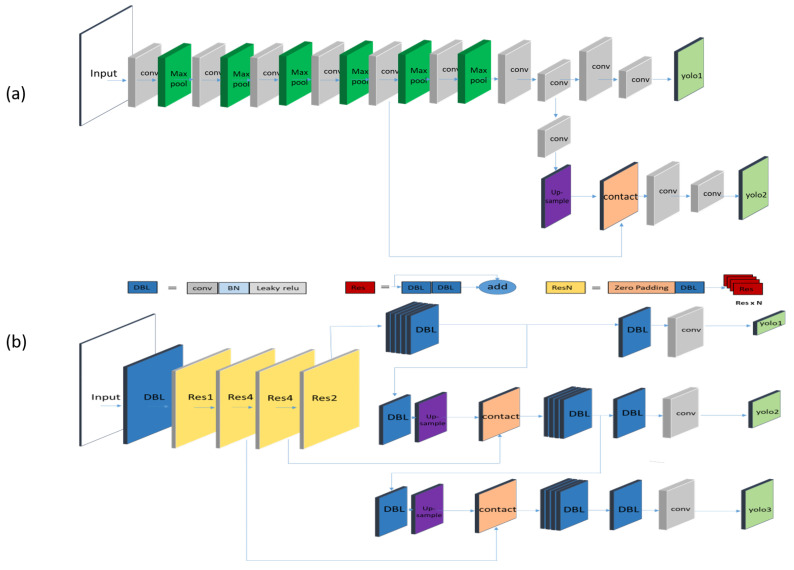
Structure of deep learning networks. (**a**) Tiny YOLOv3. (**b**) Improved YOLOv3. Tiny YOLOv3 has a simple structure with a backbone that consists of conv and max pool, while improved YOLOv3 has a more complex structure of Resnet, DBL, and FPN, which makes it more accurate.

**Figure 6:**
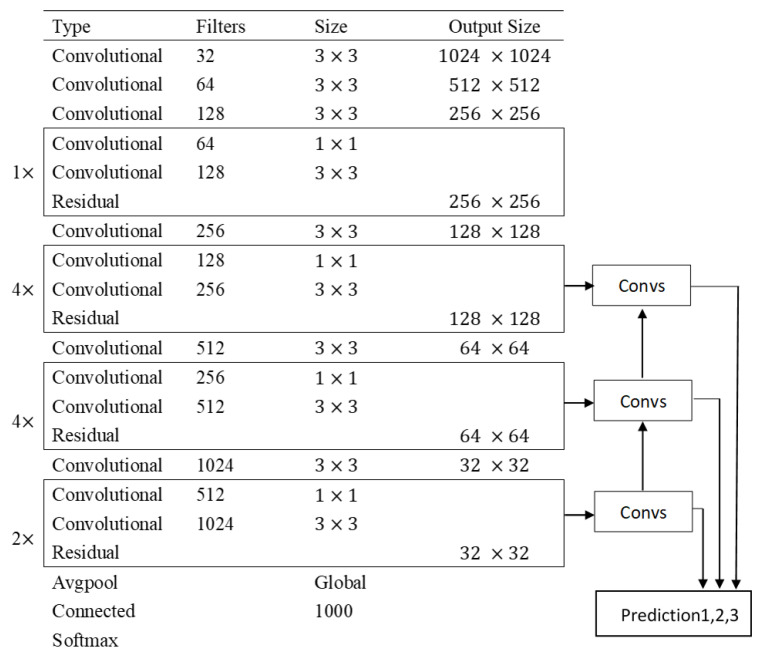
Backbone of improved YOLOv3. The backbone of improved YOLOv3 adopts the Feature Pyramid Network, so the features extracted from the upper layers are transferred to the lower layers to improve the recognition accuracy.

The structure and backbone of the improved YOLOv3 is shown in Figure 5b and Figure 6. Our test equipment has a low price that costs less than USD 100. So, the embedded device has a relatively limited processing power compared with the workstations. If the network is deployed directly on the embedded device, it is difficult to compute efficiently. Therefore, we need to accelerate the model via TensorRT.

First, we need to convert the darknet (.cfg) into the ONNX (.onnx) model. Then, TensorRT will build the inference engine, which accelerates the CNN network of improved YOLOv3. The TensorRT will take the following steps to improve the inference speed: (1) Precision calibration. Deep neural networks need high precision data to ensure accuracy during the training step, but the data precision can be moderately reduced during the inference process. So, we improve the inference speed by decreasing the data type from float32 to FP16. (2) Layer fusion. TensorRT will fuse the structure of deep neural networks. For example, it will fuse the conv, BN, and relu into one layer, so no more separate calculations are performed on contact layer, which can significantly reduce the data transfer time. (3) Multi-stream execution. TensorRT will perform parallel computation on different branches with the same input and dynamically optimize the memory according to batch size, which effectively reduces the transmission time. With the above optimizations, the data transfer efficiency and computation speed of the model can be greatly improved so that the model can perform inference at a rate of more than 1 frame per second (FPS) on a jetson nano.

### 2.8. Comparative Methods

We choose the simple threshold, comprehensive threshold, and tiny YOLOv3, which are commonly used in the field of BCC, as the comparative methods. Furthermore, we adopt the human count result as the gold standard. Simple threshold segmentation firstly adopts the cv2.cvtColor() function to change the input image into the grayscale mode, then uses the cv2.threshold() function to segment the grayscale image. Since the contrast between target and background is low, the segmentation effect of the automatic threshold value is relatively poor, so we need to manually determine and adjust the appropriate threshold value. After the threshold value is determined, the cv2.threshold() function will regard the pixels below the threshold value as background and the pixels above the threshold value as valid targets. Since simple threshold segmentation treats every continuous area larger than the threshold value as the effective target, it is very susceptible to noise interference.

Comprehensive threshold segmentation introduces the size filtering function on the basis of simple threshold segmentation. By using the Cv2.findContours() function, the comprehensive threshold calculates the circular contour of the simple threshold segmentation result, which could be used for classifying the radius and area. Then, we need to adjust and set the min_area and max_area manually. Finally, only the targets that are among the range of (min_area, max_area) will be regarded as effective. Therefore, comprehensive threshold segmentation can filter out the interference of small noise effectively, but bacterial colonies have a large number of overlapping targets that are difficult to process by threshold segmentation, so the accuracy rate of the comprehensive threshold is still low.

Due to the computing power limitation of edge computing devices, tiny YOLOv3 is one of the few deep neural networks that can be deployed on a jetson nano with relatively good performance. The structure of tiny YOLOv3 is shown in Figure 5a. Tiny YOLOv3 removes the residual layers and some feature layers, and it only retains a backbone of 6-layer conv+max with 2 independent prediction branches that own sizes of 13 ∗ 13 and 26 ∗ 26 to extract features and make predictions. Tiny YOLOv3 greatly decreases the network depth and reduces the performance requirements of computing devices, but it correspondingly sacrifices the accuracy of feature extraction, so the accuracy rate is inferior to improved YOLOv3.

## 3. Results and Discussion

### 3.1. Results Comparison

**Figure 7:**
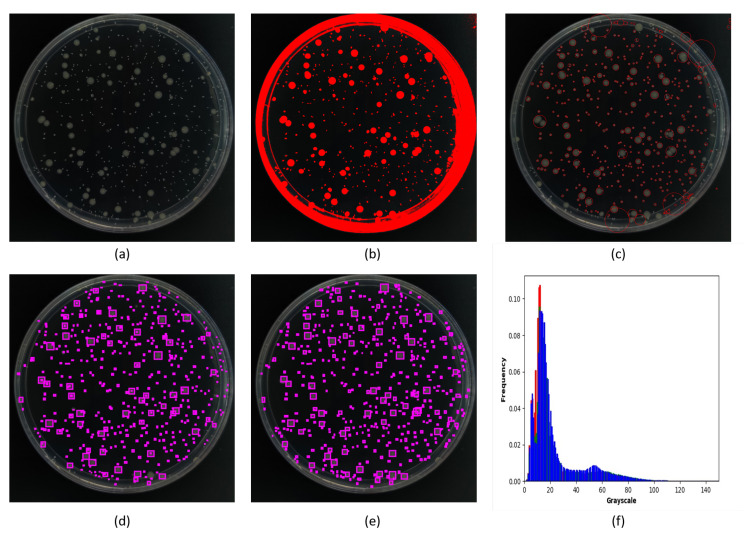
The recognition result comparison. (**a**) Original image. (**b**) Simple threshold segmentation. (**c**) Comprehensive threshold segmentation. (**d**) Tiny YOLOv3. (**e**) Improved YOLOv3. (**f**) Gray level histogram. Simple threshold has the most noise and false-positive results due to the small difference in gray value between the colony and background; comprehensive threshold segmentation reduces noise interference but has more false-negative results; tiny YOLOv3 has a large improvement in accuracy but is less effective for small targets; improved YOLOv3 has optimal results.

Table 1 and Figure 7 show the test results of different algorithms for low contrast bacterial colony images. The validation dataset contains a total of 60 images, and we adopted the sampling survey strategy to verify, which took out ten images randomly then calculated the accuracy with human measurement results as the gold standard. In Table 1, True Positive (TP) represents the number of positive targets that are correctly identified as positive; False Positive (FP) represents the number of negative targets that are incorrectly identified as positive; False Negative (FN) represents the number of positive targets that are incorrectly identified as negative; True Negative (TN) represents the number of negative targets that are correctly identified as negative; Average Accuracy (ACC) represents the average percentage of positive and negative targets that are correctly identified; True Positive Rate (TPR) represents the percentage of positive targets correctly identified as positive; False Negative Rate (FNR) represents the percentage of positive targets incorrectly identified as negative [32,33]; Detection Time (DT) represents the average processing time for each image. In our experimental results, since TN represents the number of pixels that belong to the background, which is an unquantifiable and unnecessary parameter, the TN is defaulted to 0 [34]. The human reference represents the results of the manual counting method for the colony images. Additionally, the formulas used are calculated as follows: FNR=FN/(TP+FN), ACC=(TN+TP)/(TN+TP+FN+FP),TPR=TP/(TP+FN). Since there is no True Negative situation in our samples, FN defaults to 0.

Among the three contrast methods, the comprehensive threshold and simple threshold belong to traditional algorithms, and they are the most commonly used methods for bacterial colony counting. However, they face difficulty when dealing with overlap and edge targets. Therefore, the accuracy of the traditional algorithms is not satisfying. The simple threshold is highly susceptible to small noise interference and generates a large number of false-positive targets, so its accuracy is only 4.4%. The comprehensive threshold was based on the simple threshold, and it added the size selection function. So, most small noise could be effectively removed, but the accuracy of the comprehensive threshold was still as low as 65% due to the phenomenon of adhesion and blurring of contours between bacterial colonies. For these complex targets, it is difficult for traditional algorithms to distinguish them effectively. For example, if there are multiple adhering or overlapping targets, traditional algorithms usually incorrectly consider them as the same target, thus affecting the accuracy.

As for tiny YOLOv3, it is one of the few lightweight deep learning networks that can be deployed on a jetson nano directly; it improved the accuracy to 85% compared with traditional algorithms. However, due to its shallow network depth, the recognition rate for edge targets and overlapping targets is relatively poor when compared with improved YOLOv3. On the contrary, the improved YOLOv3 proposed in this paper adopted the FPN structure such as Figure 6 shows, which will reverse the features extracted by the high-level convolution network to the lower-level convolution network, thus allowing a trade-off between speed and accuracy. For example, there are targets of different sizes in the bacterial colony images, the area of dotted bacterial colonies and circular bacterial colonies differ greatly, so the improved YOLOv3 can recognize large circular bacterial colonies through the shallow layers and the 13 ∗ 13 feature map, as well as recognize small dotted bacterial colonies through the deep layers and the 52 ∗ 52 feature map, which effectively improves the detection efficiency. Improved YOLOv3 not only ensured feasibility on a jetson nano but also retained the detection speed, which decreased the FNR to 2% and increased the accuracy to over 97% at the processing speed of more than 1 FPS. The manual counting method is the gold standard that has the highest accuracy, but it also takes the most time to detect. The improved YOLOv3 has comparable accuracy to the manual counting method, while detection time is greatly reduced.

### 3.2. Discussion

The principle of the simple threshold is to divide the image as valid and background parts by the threshold value, where the pixel gray value below the threshold is set to be 0, and the part above the threshold is set to be 1. This method is susceptible to noise interference, and it is also difficult to calculate the targets on the edge of the culture dish and overlap situation. Therefore, it is only applicable to pure pictures without noise and owns the lowest accuracy among all methods. The comprehensive threshold is an upgraded version of the simple threshold. It uses the findContours() function for calculating the radius of each target on the basis of the simple threshold segmentation, thus adding a size-based filtering function that can effectively reduce the interference of small noise. However, the comprehensive threshold is also unable to deal with overlap and adherent situations, and therefore the accuracy rate can only reach about 65%. Additionally, tiny YOLOv3 is a lightweight deep neural network, which is able to effectively identify more of the overlapping and adhering bacterial colonies and significantly improve the performance compared with traditional methods. However, since the structure of tiny YOLOv3 is relatively simple, the recognition performance for edge targets and extremely small targets is not as good as improved YOLOv3, so the accuracy rate is around 85%. In addition, traditional deep learning algorithms usually require at least hundreds of raw data to complete the effective training of deep neural networks. The adequate number of training datasets is one of the most effective methods to avoid the over-fitting problem of the deep learning models. Our method can effectively change the image structure by the data augmentation method, which can make the augmented images be regarded as a new training image for the deep neural network, and thus can reduce the original data requirement to less than 10. Compared with other traditional neural networks that require at least hundreds of training datasets [20,35,36], our method reduces the data collection cost by more than 90% while maintaining a high accuracy rate.

## 4. Conclusions

BCC plays a vital role in water contamination monitoring, food sample testing, and biological experiments. With the widening of application scenarios, accurate on-site testing is becoming important, daily, for BCC, and the three most important challenges of it are accuracy, data shortage, and portability. Currently, many labs and companies still adopt the manual counting method for BCC; this is because BCC images often have low contrast and overlap situations, making them difficult to be accurately counted. The commonly used traditional algorithms such as simple threshold and comprehensive threshold often require special color Petri dishes or professional photographic devices to enhance the contrast between bacterial colonies and the background, but these devices will increase the cost of the BCC. The development of deep learning brings new possibilities for BCC. However, general deep neural networks usually require a large number of training data and need to be deployed on professional workstations, which face difficulty in meeting the portability of the on-site testing and have a high cost of data collection. In this paper, we propose a new few-shot learning method that consists of improved YOLOv3 and RCTA to solve the above problems. This method enables us to train a network with the detection accuracy of over 97% on a jetson nano by only five raw data. RCTA can effectively augment the original training data over 300 times and save the annotation time over 80%, which greatly reduces the cost of data collection and annotation. Improved YOLOv3 can be employed on an embedded device that has low cost and achieves a high detection accuracy, which meets the portability and precision requirement of on-site testing.

Compared with traditional algorithms, improved YOLOv3 greatly optimized the detection accuracy for complex targets such as overlap and adherent, which decreased the FNR from 32% to 1.5% and increased the ACC from 64% to 97%, respectively. Additionally, if compared with one of the most widely used deep neural networks for embedded devices, tiny YOLOv3, our method decreased the FNR by over 6% and increased the average accuracy by over 10%. Moreover, our few-show learning strategy is able to train the deep learning networks with less than ten raw data, which is an amount of data that is difficult for any traditional neural network to train effectively. Furthermore, our model can achieve an inference rate of more than 1 FPS on edge computing devices after acceleration, which brings more possibilities for accurate on-site testing in the field of BCC.

## Figures and Tables

**Table 1:** Performance comparison. This table compares the performance of the five methods for bacterial colony detection. Human reference is the manual count result, which is used as the gold standard; ACC represents the average accuracy; TPR represents the percentage of colonies that are correctly identified; FNR represents the percentage of colonies that are incorrectly identified as background; DT(s) represents the average detection time in seconds for each image.

Method	TP	FP	FN	ACC	TPR	FNR	DT(s)
Human reference	4898	0	0	100%	100%	0%	257.84
Simple threshold	3605	77,484	1293	4.4%	73.8%	26.2%	0.17
Comprehensive threshold	3327	279	1571	64.3%	67.9%	32.1%	0.26
Tiny YOLOv3	4489	321	409	85.9%	91.6%	8.4%	0.50
Improved YOLOv3	4826	58	72	97.4%	98.5%	1.5%	0.89

## Data Availability

The data used in this paper can be found at: https://hkustconnect-my.sharepoint.com/:f:/g/personal/bzhangay_connect_ust_hk/Eoi0CM3IB0pMgXY__KDLM88BYp28duOeNvklJW5nmlQXPw?e=DXGODJ, accessed on 17 December 2021. The algorithm we used can be download from: https://hkustconnect-my.sharepoint.com/:f:/g/personal/bzhangay_connect_ust_hk/Er7C1SUw4LhHlnP2mJgICW4B9D-3R5Frm3Q__cIeYv2tpg?e=noCHdS, accessed on 22 November 2021.

## Abbreviations

The following abbreviations are used in this manuscript:

BCC	Bacterial Colony Counting
CNN	Convolutional Neural Network
FPN	Feature Pyramid Network
RCTA	Random Cover Targets Algorithm
YOLOv3	You Only Look Once version 3
FNR	False Negative Rate
TSB	Tryptic Soy Broth
PCA	Plate Count Agar
FPS	Frame Per Second
CFU	Colony-forming units
